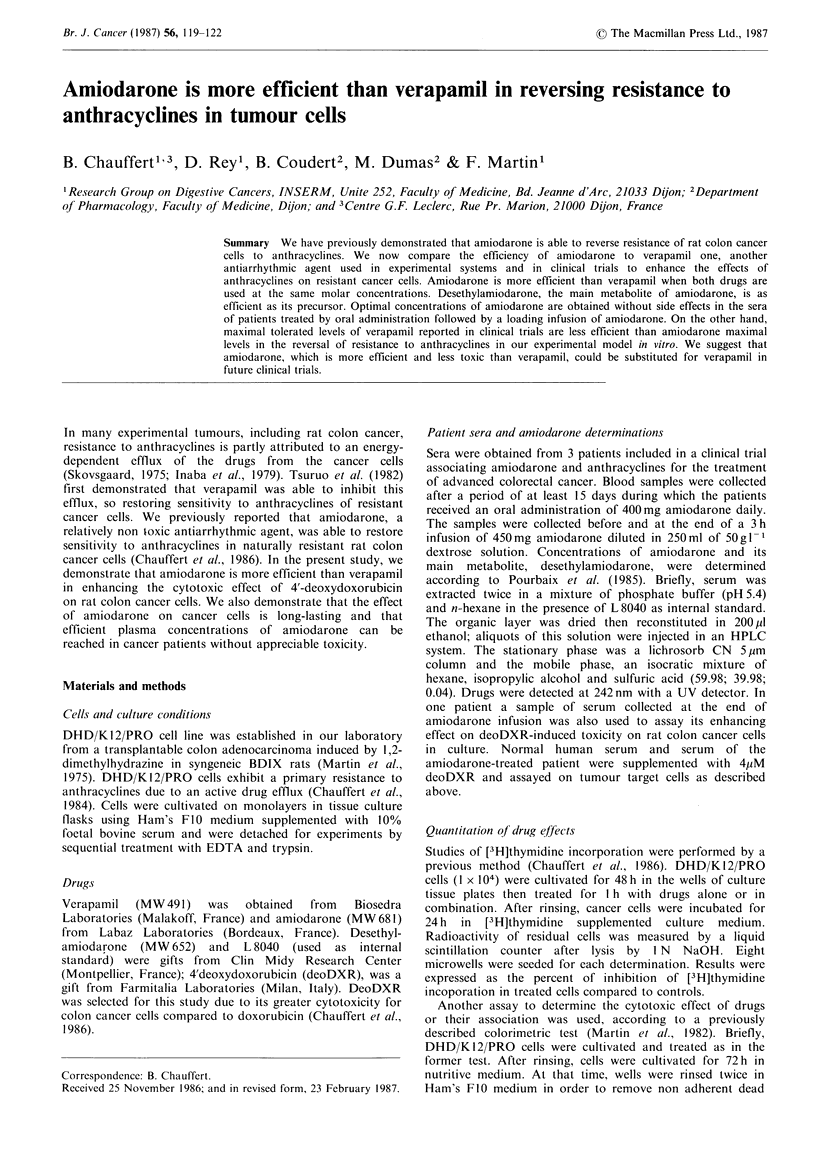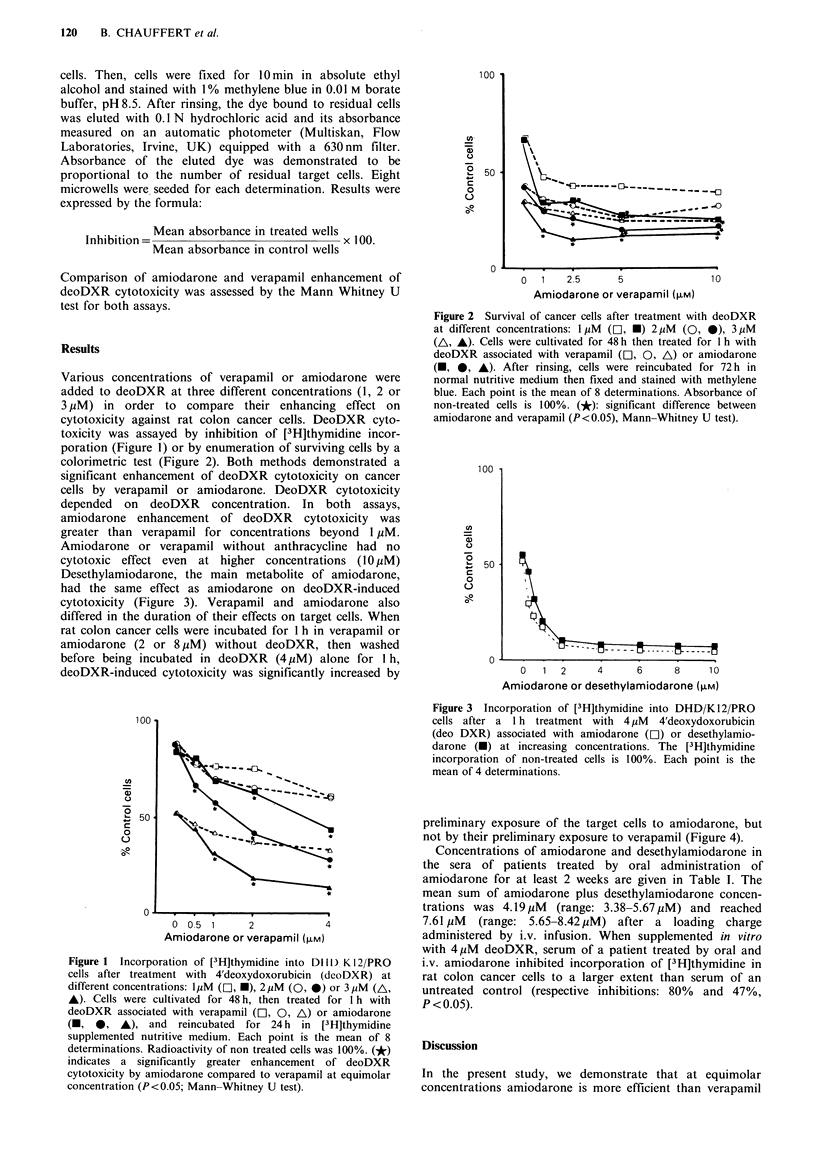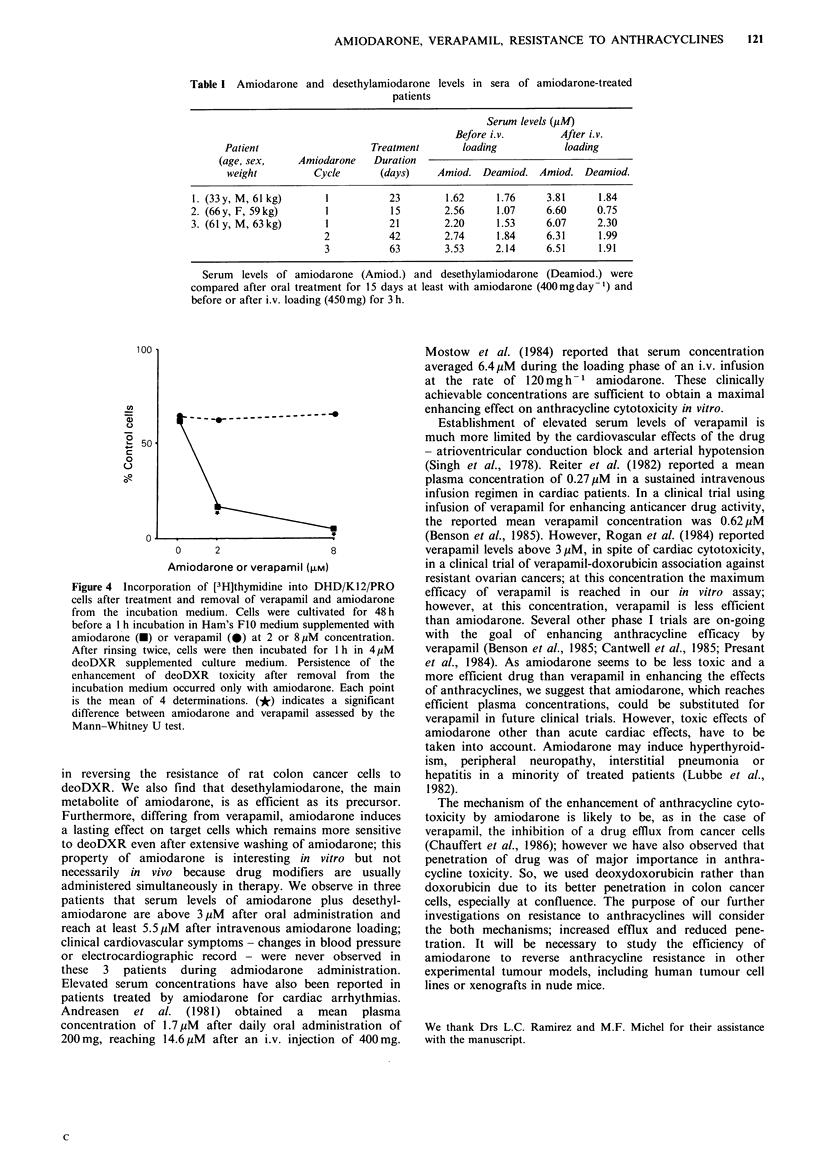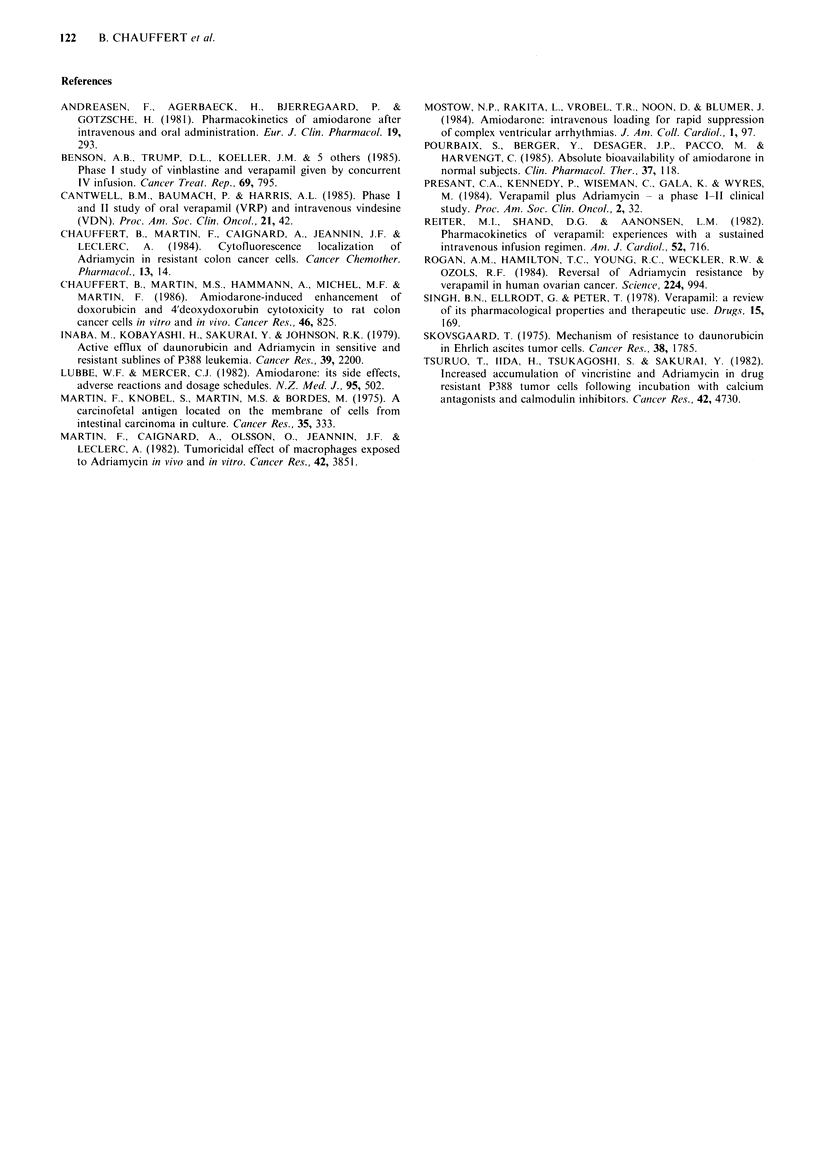# Amiodarone is more efficient than verapamil in reversing resistance to anthracyclines in tumour cells.

**DOI:** 10.1038/bjc.1987.167

**Published:** 1987-08

**Authors:** B. Chauffert, D. Rey, B. Coudert, M. Dumas, F. Martin

**Affiliations:** INSERM, Unite 252, Faculty of Medicine, Dijon, France.

## Abstract

We have previously demonstrated that amiodarone is able to reverse resistance of rat colon cancer cells to anthracyclines. We now compare the efficiency of amiodarone to verapamil one, another antiarrhythmic agent used in experimental systems and in clinical trials to enhance the effects of anthracyclines on resistant cancer cells. Amiodarone is more efficient than verapamil when both drugs are used at the same molar concentrations. Desethylamiodarone, the main metabolite of amiodarone, is as efficient as its precursor. Optimal concentrations of amiodarone are obtained without side effects in the sera of patients treated by oral administration followed by a loading infusion of amiodarone. On the other hand, maximal tolerated levels of verapamil reported in clinical trials are less efficient than amiodarone maximal levels in the reversal of resistance to anthracyclines in our experimental model in vitro. We suggest that amiodarone, which is more efficient and less toxic than verapamil, could be substituted for verapamil in future clinical trials.


Br. J. Cancer (1987) 56, 119–122

© The Macmillan Press Ltd., 1987

### Amiodarone is more efficient than verapamil in reversing resistance to anthracyclines in tumour cells

B. Chauffert[1,3], D. Rey[1], B. Coudert[2], M. Dumas[2] & F. Martin[1]

[1]*Research Group on Digestive Cancers, INSERM, Unite 252, Faculty of Medicine, Bd. Jeanne d'Arc, 21033 Dijon;* [2]*Department of Pharmacology, Faculty of Medicine, Dijon; and* [3]*Centre G.F. Leclerc, Rue Pr. Marion, 21000 Dijon, France*

**Summary** We have previously demonstrated that amiodarone is able to reverse resistance of rat colon cancer cells to anthracyclines. We now compare the efficiency of amiodarone to verapamil one, another antiarrhythmic agent used in experimental systems and in clinical trials to enhance the effects of anthracyclines on resistant cancer cells. Amiodarone is more efficient than verapamil when both drugs are used at the same molar concentrations. Desethylamiodarone, the main metabolite of amiodarone, is as efficient as its precursor. Optimal concentrations of amiodarone are obtained without side effects in the sera of patients treated by oral administration followed by a loading infusion of amiodarone. On the other hand, maximal tolerated levels of verapamil reported in clinical trials are less efficient than amiodarone maximal levels in the reversal of resistance to anthracyclines in our experimental model *in vitro*. We suggest that amiodarone, which is more efficient and less toxic than verapamil, could be substituted for verapamil in future clinical trials.

In many experimental tumours, including rat colon cancer, resistance to anthracyclines is partly attributed to an energy-dependent efflux of the drugs from the cancer cells (Skovsgaard, 1975; Inaba *et al.*, 1979). Tsuruo *et al.* (1982) first demonstrated that verapamil was able to inhibit this efflux, so restoring sensitivity to anthracyclines of resistant cancer cells. We previously reported that amiodarone, a relatively non toxic antiarrhythmic agent, was able to restore sensitivity to anthracyclines in naturally resistant rat colon cancer cells (Chauffert *et al.*, 1986). In the present study, we demonstrate that amiodarone is more efficient than verapamil in enhancing the cytotoxic effect of 4'-deoxydoxorubicin on rat colon cancer cells. We also demonstrate that the effect of amiodarone on cancer cells is long-lasting and that efficient plasma concentrations of amiodarone can be reached in cancer patients without appreciable toxicity.

### Materials and methods

### *Cells and culture conditions*

DHD/K12/PRO cell line was established in our laboratory from a transplantable colon adenocarcinoma induced by 1,2-dimethylhydrazine in syngeneic BDIX rats (Martin *et al.*, 1975). DHD/K12/PRO cells exhibit a primary resistance to anthracyclines due to an active drug efflux (Chauffert *et al.*, 1984). Cells were cultivated on monolayers in tissue culture flasks using Ham's F10 medium supplemented with 10% foetal bovine serum and were detached for experiments by sequential treatment with EDTA and trypsin.

### *Drugs*

Verapamil (MW491) was obtained from Biosedra Laboratories (Malakoff, France) and amiodarone (MW681) from Labaz Laboratories (Bordeaux, France). Desethylamiodarone (MW652) and L8040 (used as internal standard) were gifts from Clin Midy Research Center (Montpellier, France); 4'deoxydoxorubicin (deoDXR), was a gift from Farmitalia Laboratories (Milan, Italy). DeoDXR was selected for this study due to its greater cytotoxicity for colon cancer cells compared to doxorubicin (Chauffert *et al.*, 1986).

Correspondence: B. Chauffert.
Received 25 November 1986; and in revised form, 23 February 1987.

### *Patient sera and amiodarone determinations*

Sera were obtained from 3 patients included in a clinical trial associating amiodarone and anthracyclines for the treatment of advanced colorectal cancer. Blood samples were collected after a period of at least 15 days during which the patients received an oral administration of 400 mg amiodarone daily. The samples were collected before and at the end of a 3 h infusion of 450 mg amiodarone diluted in 250 ml of $50\,g\,l^{-1}$ dextrose solution. Concentrations of amiodarone and its main metabolite, desethylamiodarone, were determined according to Pourbaix *et al.* (1985). Briefly, serum was extracted twice in a mixture of phosphate buffer (pH 5.4) and *n*-hexane in the presence of L8040 as internal standard. The organic layer was dried then reconstituted in $200\,\mu l$ ethanol; aliquots of this solution were injected in an HPLC system. The stationary phase was a lichrosorb CN $5\,\mu m$ column and the mobile phase, an isocratic mixture of hexane, isopropylic alcohol and sulfuric acid (59.98; 39.98; 0.04). Drugs were detected at 242 nm with a UV detector. In one patient a sample of serum collected at the end of amiodarone infusion was also used to assay its enhancing effect on deoDXR-induced toxicity on rat colon cancer cells in culture. Normal human serum and serum of the amiodarone-treated patient were supplemented with $4\mu M$ deoDXR and assayed on tumour target cells as described above.

### *Quantitation of drug effects*

Studies of [$^3$H]thymidine incorporation were performed by a previous method (Chauffert *et al.*, 1986). DHD/K12/PRO cells ($1 \times 10^4$) were cultivated for 48 h in the wells of culture tissue plates then treated for 1 h with drugs alone or in combination. After rinsing, cancer cells were incubated for 24 h in [$^3$H]thymidine supplemented culture medium. Radioactivity of residual cells was measured by a liquid scintillation counter after lysis by 1 N NaOH. Eight microwells were seeded for each determination. Results were expressed as the percent of inhibition of [$^3$H]thymidine incoporation in treated cells compared to controls.

Another assay to determine the cytotoxic effect of drugs or their association was used, according to a previously described colorimetric test (Martin *et al.*, 1982). Briefly, DHD/K12/PRO cells were cultivated and treated as in the former test. After rinsing, cells were cultivated for 72 h in nutritive medium. At that time, wells were rinsed twice in Ham's F10 medium in order to remove non adherent dead cells. Then, cells were fixed for 10 min in absolute ethyl alcohol and stained with 1% methylene blue in 0.01 M borate buffer, pH 8.5. After rinsing, the dye bound to residual cells was eluted with 0.1 N hydrochloric acid and its absorbance measured on an automatic photometer (Multiskan, Flow Laboratories, Irvine, UK) equipped with a 630 nm filter. Absorbance of the eluted dye was demonstrated to be proportional to the number of residual target cells. Eight microwells were seeded for each determination. Results were expressed by the formula:

$$\text{Inhibition} = \frac{\text{Mean absorbance in treated wells}}{\text{Mean absorbance in control wells}} \times 100.$$

Comparison of amiodarone and verapamil enhancement of deoDXR cytotoxicity was assessed by the Mann Whitney U test for both assays.

### Results

Various concentrations of verapamil or amiodarone were added to deoDXR at three different concentrations (1, 2 or 3 $\mu$M) in order to compare their enhancing effect on cytotoxicity against rat colon cancer cells. DeoDXR cytotoxicity was assayed by inhibition of [$^3$H]thymidine incorporation (Figure 1) or by enumeration of surviving cells by a colorimetric test (Figure 2). Both methods demonstrated a significant enhancement of deoDXR cytotoxicity on cancer cells by verapamil or amiodarone. DeoDXR cytotoxicity depended on deoDXR concentration. In both assays, amiodarone enhancement of deoDXR cytotoxicity was greater than verapamil for concentrations beyond 1 $\mu$M. Amiodarone or verapamil without anthracycline had no cytotoxic effect even at higher concentrations (10 $\mu$M) Desethylamiodarone, the main metabolite of amiodarone, had the same effect as amiodarone on deoDXR-induced cytotoxicity (Figure 3). Verapamil and amiodarone also differed in the duration of their effects on target cells. When rat colon cancer cells were incubated for 1 h in verapamil or amiodarone (2 or 8 $\mu$M) without deoDXR, then washed before being incubated in deoDXR (4 $\mu$M) alone for 1 h, deoDXR-induced cytotoxicity was significantly increased by

**Figure 1** Incorporation of [$^3$H]thymidine into DHD/K12/PRO cells after treatment with 4'deoxydoxorubicin (deoDXR) at different concentrations: 1 $\mu$M (□, ■), 2 $\mu$M (○, ●) or 3 $\mu$M (△, ▲). Cells were cultivated for 48 h, then treated for 1 h with deoDXR associated with verapamil (□, ○, △) or amiodarone (■, ●, ▲), and reincubated for 24 h in [$^3$H]thymidine supplemented nutritive medium. Each point is the mean of 8 determinations. Radioactivity of non treated cells was 100%. (★) indicates a significantly greater enhancement of deoDXR cytotoxicity by amiodarone compared to verapamil at equimolar concentration ($P<0.05$; Mann–Whitney U test).

**Figure 2** Survival of cancer cells after treatment with deoDXR at different concentrations: 1 $\mu$M (□, ■) 2 $\mu$M (○, ●), 3 $\mu$M (△, ▲). Cells were cultivated for 48 h then treated for 1 h with deoDXR associated with verapamil (□, ○, △) or amiodarone (■, ●, ▲). After rinsing, cells were reincubated for 72 h in normal nutritive medium then fixed and stained with methylene blue. Each point is the mean of 8 determinations. Absorbance of non-treated cells is 100%. (★): significant difference between amiodarone and verapamil ($P<0.05$), Mann–Whitney U test).

**Figure 3** Incorporation of [$^3$H]thymidine into DHD/K12/PRO cells after a 1 h treatment with 4 $\mu$M 4'deoxydoxorubicin (deo DXR) associated with amiodarone (□) or desethylamiodarone (■) at increasing concentrations. The [$^3$H]thymidine incorporation of non-treated cells is 100%. Each point is the mean of 4 determinations.

preliminary exposure of the target cells to amiodarone, but not by their preliminary exposure to verapamil (Figure 4).

Concentrations of amiodarone and desethylamiodarone in the sera of patients treated by oral administration of amiodarone for at least 2 weeks are given in Table I. The mean sum of amiodarone plus desethylamiodarone concentrations was 4.19 $\mu$M (range: 3.38–5.67 $\mu$M) and reached 7.61 $\mu$M (range: 5.65–8.42 $\mu$M) after a loading charge administered by i.v. infusion. When supplemented *in vitro* with 4 $\mu$M deoDXR, serum of a patient treated by oral and i.v. amiodarone inhibited incorporation of [$^3$H]thymidine in rat colon cancer cells to a larger extent than serum of an untreated control (respective inhibitions: 80% and 47%, $P<0.05$).

### Discussion

In the present study, we demonstrate that at equimolar concentrations amiodarone is more efficient than verapamil

**Table I** Amiodarone and desethylamiodarone levels in sera of amiodarone-treated patients

| *Patient (age, sex, weight* | *Amiodarone Cycle* | *Treatment Duration (days)* | *Serum levels (μM)* *Before i.v. loading* | | *After i.v. loading* | |
|---|---|---|---|---|---|---|
| | | | *Amiod.* | *Deamiod.* | *Amiod.* | *Deamiod.* |
| 1. (33 y, M, 61 kg) | 1 | 23 | 1.62 | 1.76 | 3.81 | 1.84 |
| 2. (66 y, F, 59 kg) | 1 | 15 | 2.56 | 1.07 | 6.60 | 0.75 |
| 3. (61 y, M, 63 kg) | 1 | 21 | 2.20 | 1.53 | 6.07 | 2.30 |
| | 2 | 42 | 2.74 | 1.84 | 6.31 | 1.99 |
| | 3 | 63 | 3.53 | 2.14 | 6.51 | 1.91 |

Serum levels of amiodarone (Amiod.) and desethylamiodarone (Deamiod.) were compared after oral treatment for 15 days at least with amiodarone (400 mg day$^{-1}$) and before or after i.v. loading (450 mg) for 3 h.

**Figure 4** Incorporation of [$^3$H]thymidine into DHD/K12/PRO cells after treatment and removal of verapamil and amiodarone from the incubation medium. Cells were cultivated for 48 h before a 1 h incubation in Ham's F10 medium supplemented with amiodarone (■) or verapamil (●) at 2 or 8 μM concentration. After rinsing twice, cells were then incubated for 1 h in 4 μM deoDXR supplemented culture medium. Persistence of the enhancement of deoDXR toxicity after removal from the incubation medium occurred only with amiodarone. Each point is the mean of 4 determinations. (★) indicates a significant difference between amiodarone and verapamil assessed by the Mann–Whitney U test.

in reversing the resistance of rat colon cancer cells to deoDXR. We also find that desethylamiodarone, the main metabolite of amiodarone, is as efficient as its precursor. Furthermore, differing from verapamil, amiodarone induces a lasting effect on target cells which remains more sensitive to deoDXR even after extensive washing of amiodarone; this property of amiodarone is interesting *in vitro* but not necessarily *in vivo* because drug modifiers are usually administered simultaneously in therapy. We observe in three patients that serum levels of amiodarone plus desethylamiodarone are above 3 μM after oral administration and reach at least 5.5 μM after intravenous amiodarone loading; clinical cardiovascular symptoms – changes in blood pressure or electrocardiographic record – were never observed in these 3 patients during admiodarone administration. Elevated serum concentrations have also been reported in patients treated by amiodarone for cardiac arrhythmias. Andreasen *et al.* (1981) obtained a mean plasma concentration of 1.7 μM after daily oral administration of 200 mg, reaching 14.6 μM after an i.v. injection of 400 mg. Mostow *et al.* (1984) reported that serum concentration averaged 6.4 μM during the loading phase of an i.v. infusion at the rate of 120 mg h$^{-1}$ amiodarone. These clinically achievable concentrations are sufficient to obtain a maximal enhancing effect on anthracycline cytotoxicity *in vitro*.

Establishment of elevated serum levels of verapamil is much more limited by the cardiovascular effects of the drug – atrioventricular conduction block and arterial hypotension (Singh *et al.*, 1978). Reiter *et al.* (1982) reported a mean plasma concentration of 0.27 μM in a sustained intravenous infusion regimen in cardiac patients. In a clinical trial using infusion of verapamil for enhancing anticancer drug activity, the reported mean verapamil concentration was 0.62 μM (Benson *et al.*, 1985). However, Rogan *et al.* (1984) reported verapamil levels above 3 μM, in spite of cardiac cytotoxicity, in a clinical trial of verapamil-doxorubicin association against resistant ovarian cancers; at this concentration the maximum efficacy of verapamil is reached in our *in vitro* assay; however, at this concentration, verapamil is less efficient than amiodarone. Several other phase I trials are on-going with the goal of enhancing anthracycline efficacy by verapamil (Benson *et al.*, 1985; Cantwell *et al.*, 1985; Presant *et al.*, 1984). As amiodarone seems to be less toxic and a more efficient drug than verapamil in enhancing the effects of anthracyclines, we suggest that amiodarone, which reaches efficient plasma concentrations, could be substituted for verapamil in future clinical trials. However, toxic effects of amiodarone other than acute cardiac effects, have to be taken into account. Amiodarone may induce hyperthyroidism, peripheral neuropathy, interstitial pneumonia or hepatitis in a minority of treated patients (Lubbe *et al.*, 1982).

The mechanism of the enhancement of anthracycline cytotoxicity by amiodarone is likely to be, as in the case of verapamil, the inhibition of a drug efflux from cancer cells (Chauffert *et al.*, 1986); however we have also observed that penetration of drug was of major importance in anthracycline toxicity. So, we used deoxydoxorubicin rather than doxorubicin due to its better penetration in colon cancer cells, especially at confluence. The purpose of our further investigations on resistance to anthracyclines will consider the both mechanisms; increased efflux and reduced penetration. It will be necessary to study the efficiency of amiodarone to reverse anthracycline resistance in other experimental tumour models, including human tumour cell lines or xenografts in nude mice.

We thank Drs L.C. Ramirez and M.F. Michel for their assistance with the manuscript.

### References

ANDREASEN, F., AGERBAECK, H., BJERREGAARD, P. & GOTZSCHE, H. (1981). Pharmacokinetics of amiodarone after intravenous and oral administration. *Eur. J. Clin. Pharmacol.* **19**, 293.

BENSON, A.B., TRUMP, D.L., KOELLER, J.M. & 5 others (1985). Phase I study of vinblastine and verapamil given by concurrent IV infusion. *Cancer Treat. Rep.*, **69**, 795.

CANTWELL, B.M., BAUMACH, P. & HARRIS, A.L. (1985). Phase I and II study of oral verapamil (VRP) and intravenous vindesine (VDN). *Proc. Am. Soc. Clin. Oncol.*, **21**, 42.

CHAUFFERT, B., MARTIN, F., CAIGNARD, A., JEANNIN, J.F. & LECLERC, A. (1984). Cytofluorescence localization of Adriamycin in resistant colon cancer cells. *Cancer Chemother. Pharmacol.*, **13**, 14.

CHAUFFERT, B., MARTIN, M.S., HAMMANN, A., MICHEL, M.F. & MARTIN, F. (1986). Amiodarone-induced enhancement of doxorubicin and 4'deoxydoxorubin cytotoxicity to rat colon cancer cells *in vitro* and *in vivo*. *Cancer Res.*, **46**, 825.

INABA, M., KOBAYASHI, H., SAKURAI, Y. & JOHNSON, R.K. (1979). Active efflux of daunorubicin and Adriamycin in sensitive and resistant sublines of P388 leukemia. *Cancer Res.*, **39**, 2200.

LUBBE, W.F. & MERCER, C.J. (1982). Amiodarone: its side effects, adverse reactions and dosage schedules. *N.Z. Med. J.*, **95**, 502.

MARTIN, F., KNOBEL, S., MARTIN, M.S. & BORDES, M. (1975). A carcinofetal antigen located on the membrane of cells from intestinal carcinoma in culture. *Cancer Res.*, **35**, 333.

MARTIN, F., CAIGNARD, A., OLSSON, O., JEANNIN, J.F. & LECLERC, A. (1982). Tumoricidal effect of macrophages exposed to Adriamycin *in vivo* and *in vitro*. *Cancer Res.*, **42**, 3851.

MOSTOW, N.P., RAKITA, L., VROBEL, T.R., NOON, D. & BLUMER, J. (1984). Amiodarone: intravenous loading for rapid suppression of complex ventricular arrhythmias. *J. Am. Coll. Cardiol.*, **1**, 97.

POURBAIX, S., BERGER, Y., DESAGER, J.P., PACCO, M. & HARVENGT, C. (1985). Absolute bioavailability of amiodarone in normal subjects. *Clin. Pharmacol. Ther.*, **37**, 118.

PRESANT, C.A., KENNEDY, P., WISEMAN, C., GALA, K. & WYRES, M. (1984). Verapamil plus Adriamycin – a phase I–II clinical study. *Proc. Am. Soc. Clin. Oncol.*, **2**, 32.

REITER, M.I., SHAND, D.G. & AANONSEN, L.M. (1982). Pharmacokinetics of verapamil: experiences with a sustained intravenous infusion regimen. *Am. J. Cardiol.*, **52**, 716.

ROGAN, A.M., HAMILTON, T.C., YOUNG, R.C., WECKLER, R.W. & OZOLS, R.F. (1984). Reversal of Adriamycin resistance by verapamil in human ovarian cancer. *Science*, **224**, 994.

SINGH, B.N., ELLRODT, G. & PETER, T. (1978). Verapamil: a review of its pharmacological properties and therapeutic use. *Drugs*, **15**, 169.

SKOVSGAARD, T. (1975). Mechanism of resistance to daunorubicin in Ehrlich ascites tumor cells. *Cancer Res.*, **38**, 1785.

TSURUO, T., IIDA, H., TSUKAGOSHI, S. & SAKURAI, Y. (1982). Increased accumulation of vincristine and Adriamycin in drug resistant P388 tumor cells following incubation with calcium antagonists and calmodulin inhibitors. *Cancer Res.*, **42**, 4730.